# Localization of the Serotonin Transporter in the Dog Intestine and Comparison to the Rat and Human Intestines

**DOI:** 10.3389/fvets.2021.802479

**Published:** 2022-01-05

**Authors:** Roberto Chiocchetti, Giorgia Galiazzo, Fiorella Giancola, Claudio Tagliavia, Chiara Bernardini, Monica Forni, Marco Pietra

**Affiliations:** Department of Veterinary Medical Sciences (UNI EN ISO 9001:2008), University of Bologna, Ozzano dell'Emilia, Italy

**Keywords:** enteric nervous system, myenteric plexus, SERT, submucosal plexus, 5-HT

## Abstract

Serotonin is crucial in gastrointestinal functions, including motility, sensitivity, secretion, and the inflammatory response. The serotonin transporter (SERT), responsible for serotonin reuptake and signaling termination, plays a prominent role in gastrointestinal physiology, representing a promising therapeutic target in digestive disorders. Serotonin transporter expression has been poorly investigated in veterinary medicine, under both healthy and pathological conditions, including canine chronic enteropathy, in which the serotonin metabolism seems to be altered. The aim of the present study was to determine the distribution of SERT immunoreactivity (SERT-IR) in the dog intestine and to compare the findings with those obtained in the rat and human intestines. Serotonin transporter-IR was observed in canine enterocytes, enteric neurons, *lamina propria* cells and the *tunica muscularis*. Data obtained in dogs were consistent with those obtained in rats and humans. Since the majority of the serotonin produced by the body is synthesized in the gastrointestinal tract, SERT-expressing cells may exert a role in the mechanism of serotonin reuptake.

## Introduction

Serotonin (5-hydroxytryptamine [5-HT]) is one of the most important monoamine neurotransmitters in the central (CNS) and peripheral nervous systems (1). Although 5-HT exerts a crucial role in the CNS, only 5% of the endogenous 5-HT is localized in the brain neurons (2); 95% is present in the gut where 5-HT is synthesized by the enterochromaffin cells (ECs). A small amount (up to 2%) is found in enteric neurons (mainly interneurons) in some species (3–7). Despite their small numbers, serotonergic neurons project widely throughout the enteric nervous system (ENS) and also innervate the interstitial cells of Cajal (ICC) (8, 9). Enterochromaffin cells represent the major (if not exclusive) source of 5-HT in the blood, which is taken up and conveyed by platelets throughout the bloodstream, thereby exerting its biological effects via 15 different receptor subtypes (10, 11). In gastrointestinal tract (GIT) physiology and pathophysiology, the importance of the 5-HT is due to its dual role as an intercellular signaling mucosal molecule and neurotransmitter (12, 13). In the GIT, mechanical, chemical and nervous stimuli lead to the secretion of 5-HT into the gut lumen and *lamina propria* from ECs (7). Once released by the ECs, 5-HT binds to specific 5-HT receptors located on the enteric neurons and nerve fibers, immune cells, smooth muscle cells, epithelial cells and blood vessels, and induces different responses, modulating GIT functions, such as motility, sensitivity, secretion and inflammation (1, 11, 14, 15). The multiplicity of enteric 5-HT targets and 5-HT receptors complicates ascertaining the physiological roles of 5-HT (9). Although it is well-known that exogenous serotonin potently stimulates gastrointestinal motility, the role of the endogenous serotonin released from the ECs is unclear, and is still the subject of debate. The mutation of the gene encoding the enzyme responsible for the synthesis of mucosal serotonin (i.e., tryptophan hydroxylase 1) (16, 17) does not lead to a reduction in transit *in vivo* (18). Other studies have shown that complete deletion of all endogenous serotonin from the colon does not prevent peristalsis (19, 20) or colonic migrating motor complexes (21). The aforementioned findings are in contrast with other studies which have shown that the pharmacological reduction of the 5-HT transmembrane transport is not sufficient to reset the 5-HT cellular production to zero and prevent the remaining 5-HT from possibly activating the 5-HT receptors and enhancing intestinal motility (9, 22). The most recent literature has indicated that serotonin is released from the ECs in response to contraction of the GIT (23), and that this subsequently modulates the frequency of contractile events by means of interaction with nerve processes of the myenteric plexus (MP) neurons (24, 25).

In summary, the discussion regarding the role played by 5-HT in peristalsis continues and, although knowledge regarding serotonin-peristalsis has been progressing, it seems that the functional roles of mucosal 5-HT remain unclear.

A specialized serotonin transporter (SERT), belonging to the solute carrier superfamily, is responsible for the termination of 5-HT signaling. Serotonin reuptake by the SERT consists of a mechanism associated with Na^+^ and Cl^−^ cotransport and K^+^ counter-transport (26–28). In the GIT of human and murine species, the SERT has been shown to be expressed by enterocytes, enteric nerves, and endothelial cells (29–32), and its functional activity depends on its expression at the cellular membrane level. In addition, the SERT has been identified on different types of mucosal immune cells, such as monocytes/macrophages, mast cells, B and T cells, and dendritic cells (11).

Serotonin transporter expression can be modified via phosphorylation by protein kinase C. Once phosphorylated, the transporter is internalized, resulting in a decreased amount of 5-HT reuptake (33). Serotonin transporter internalization is inhibited by the active transport of 5-HT to modulate 5-HT bioavailability at the tissue level (34). Thus, SERT expression at the intestinal level and its role in the development of GIT sensory and motor dysfunctions is a hot topic in gastroenterology. Serotonin transporter expression in the GIT has been one of the most investigated topics in different digestive disorders, such as inflammatory bowel disease (IBD) and irritable bowel syndrome (IBS), in which the 5-HT metabolism seems to be altered (31, 32, 35–37). Thus, the identification of SERT expression in the human intestine, under both healthy and pathological conditions, remains a major focus for researchers, representing a promising therapeutic target.

In the veterinary medicine context, the GIT expression and activity of 5-HT, 5-HT receptors and SERT have only been partially investigated (38–40). The localization of intestinal SERT, widely studied in humans and rodents (27, 28, 30–32, 41), may provide a basis for additional studies aimed at gaining a better understanding of the mechanisms involved in the genesis and treatment of canine chronic enteropathy (42).

The selective serotonin reuptake inhibitors (SSRIs), antidepressants, have become the largest class of medications prescribed for depression in humans. Of the SSRIs, fluoxetine has also been approved for veterinary use in treatment of canine separation anxiety (43). Although studies do not report specific signs of serotonin toxicity with regard to fluoxetine, appropriate knowledge of the anatomical distribution of the SERT in the canine GIT is necessary to better understand any undesiderable effects due to the accidental ingestion of the drug by pets.

Serotonin transporter expression has never been immunohistochemically investigated in dogs. Therefore, the aim of the present study was to determine, for the first time, the distribution of SERT immunoreactivity in the dog intestine and to compare the findings obtained in dogs with those obtained in the rat and human intestines. In addition, since little and discordant research has not yet elucidated whether the dog ENS hosts serotoninergic neurons, the present study attempted to identify the serotoninergic neurons in canine intestinal tissues.

## Materials and Methods

### Animals and Tissue Collection

Canine tissues were collected from seven dogs (Table 1) with the owner's permission. The dogs did not have any history of gastrointestinal disorders and did not show gross alterations in the gastrointestinal wall at *post-mortem* examination; they died naturally or were euthanized for humane reasons. According to Directive 2010/63/EU of the European Parliament and of the Council of 22 September 2010 regarding the protection of animals used for scientific purposes, Italian legislation (D. Lgs. n. 26/2014) does not require any approval by competent authorities or ethics committees since this study did not induce any pain or influence any therapeutic decisions.

**Table 1:** Signalment and cause of death of the dogs included in the present study.

**Dog**	**Breed**	**Sex**	**Age**	**Cause of death**
#1	Chihuahua	F	8 m	Head trauma
#2	Labrador retriever	F^S^	11 y	Acute kidney injury
#3	Belgian shepherd dog	M	11y	Thoracic disk herniation
#4	West highland white terrier	M	17 y	Intracranial neoplasia
#5	Half-breed	M^N^	11 y	Hemangiosarcoma
#6	Bergamasco shepherd	M	11y	Addison's disease
#7	German hound	M	13 y	Pulmonary edema

*F, female; F^S^ spayed female; M, male; M^N^, neutered male*.

### Gastrointestinal Dog Tissues

The entire dog intestine was removed within 1 h after each animal's death and was longitudinally opened along the mesenteric border. Specimens of the descending duodenum, ileum, and descending colon were isolated. After removal, tissue samples of the ileum (full thickness) for Western Blot (WB) validation of the anti-SERT antibody were immediately frozen in liquid nitrogen and stored at −80°C until used. Specimens for immunohistochemistry (IHC) were flushed with PBS (phosphate buffered saline, 0.15 M NaCl in 0.01 M sodium phosphate buffer, pH 7.2), gently pinned (without stretching) with brass pins on balsa wood, and fixed in 2% paraformaldehyde plus 0.2% picric acid in 0.1 M sodium phosphate buffer (pH 7.0) at 4°C overnight. After washing in PBS, tissues from the intestine were treated to obtain tangential (to the serosal surface of the tissues) (about 1.0 cm × 1.0 cm) and longitudinal (about 2.0 cm × 0.5 cm) cryosections (44). The canine tissues were also treated to obtain wholemount preparations, as described in Giancola et al. (45).

### Immunohistochemistry

In the present study, the rabbit polyclonal anti-SERT antibody (AB9726, Merk) directed against the fusion protein from the N-terminal of the rat SERT (46) was used. This antibody, which had previously been validated by Yang et al. (47) using WB analysis on rat tissues, was employed as positive control in the current study on cryosections of the rat ileum and colon, which were fixed and processed for immunohistochemistry as described above (authorization no. 112/2018-PR of 12 February 2018). In addition, wholemount preparations of the rat ileum and colon were analyzed using prevalidated immunohistochemical protocols (48). Since anti-SERT antibody reacts with human tissues (49), cryosections of biopsies of the human duodenum and descending colon were used as positive controls, after donor consent.

The immunogen used to obtain the anti-HuC/HuD antibody was the human HuC/HuD neuronal protein. The present study group had previously tested the specificity of this pan-neuronal marker on canine tissues using WB analysis (45).

The immunogen used to obtain the monoclonal anti-serotonin antibody was human 5-HT hydrochloride. The supplier indicated cross-reactivity with 5-HT in the canine tissues (39).

The antibody against the vasoactive intestinal polypeptide (VIP), designated as CURE.V55, had been generated by a very stable cell line of mouse spleen (50). Immunohistochemical studies have shown that the anti-VIP antibody stains neurons and nerve fibers in human and rat intestinal tissues as has previously been described with the polyclonal anti-VIP antiserum (#7913) developed by the same laboratory (51), and which has been used successfully on canine enteric tissues.

The specificity of the secondary antibodies was tested by applying them after omission of the primary antibodies. No stained cells could be detected after omitting the primary antibodies. In double-immunostaining protocols, control experiments were also carried out to check for the nonspecific binding of the secondary antibodies to inappropriate primary antibodies by omission of one or the other of the first stage reagents. Furthermore, incubation with two primary antibodies followed by only one secondary antibody was carried out to check for the existence of any cross-reactivity between the primary and the secondary antibodies. No evidence of non-specific binding was found.

The cryosections were hydrated in PBS and processed for immunostaining. To block non-specific binding, the sections were incubated in a solution containing 20% normal donkey serum (Colorado Serum Co., Denver, CO, USA), 1% Triton X-100 (T8787, Sigma Aldrich, MO, USA), and 1% bovine serum albumin (A-9418, Sigma Aldrich, MO, USA) in PBS for 1 h at RT. The cryosections were incubated overnight in a humid chamber at room temperature (RT) with the primary antibodies (Table 2) diluted in 1.8% NaCl in 0.01M PBS containing 0.1% sodium azide. After washing in PBS (3 x 10 min), the sections were incubated for 1 h at RT in a humid chamber with the secondary antibodies (Table 3) diluted in PBS. The cryosections were then washed in PBS (3 x 10 min) and mounted in buffered glycerol at pH 8.6 with 4',6-diamidino-2-phenylindole (DAPI; Santa Cruz Biotechnology, CA, USA). The neurons were identified with the mouse anti-HuC/HuD antibody.

**Table 2:** Details of the primary antibodies utilized on the wholemount preparations (wm) and/or cryosections (cryo).

**Primary antibody**	**Host**	**Code**	**Dilution**	**Source**
HuC/HuD	Mouse	A21271 RRID:AB_221448	1:200 wm	Thermo Fisher
Serotonin	Mouse	(5-HT-H209) RRID:AB_302212	1:250 wm 1:500 cryo	Abcam
SERT	Rabbit	AB9726 RRID:AB_612176	1:250 wm 1:500 cryo	Merck
VIP	Mouse	CURE.V55	1:5000 cryo	Gift of Prof. C. Sternini^a^

*Abcam, Cambridge, United Kingdom, Europe; Merck Millipore, Merck KGaA, Darmstadt, Germany, Europe; Thermo Fisher Scientific, Waltham, MA USA*.

a *Division of Digestive Diseases, Department Medicine, David Geffen School of Medicine, UCLA, Los Angeles, CA, United States*.

**Table 3:** Details of the secondary antibodies utilized on the wholemount preparations (wm) and/or cryosections (cryo).

**Secondary antibody**	**Host**	**Code**	**Dilution**	**Source**
Anti-mouse IgG Alexa-594	Donkey	A-21203 RRID:AB_141633	1:250 wm 1:500 cryo	Thermo Fisher
Anti-mouse IgG Alexa-488	Donkey	A-21202 RRID:AB_141607	1:250 wm 1:500 cryo	Thermo Fisher
Anti-rabbit IgG Alexa-488	Donkey	A-21206 RRID:AB_2535792	1:500 wm 1:1000 cryo	Thermo Fisher
Anti-rabbit IgG Alexa-594	Donkey	ab150076 RRID:AB_2782993	1:600	Abcam

*Abcam, Cambridge, United Kingdom, Europe; Thermo Fisher Scientific, Waltham, MA, United States*.

### Analysis of the Sections

The preparations were examined using a Nikon Eclipse Ni microscope equipped with the appropriate filter cubes to differentiate the fluorochromes employed. The images were recorded using a Nikon DS-Qi1Nc digital camera and NIS Elements software BR 4.20.01 (Nikon Instruments Europe BV, Amsterdam, Netherlands). Slight adjustments to contrast and brightness were made using Corel Photo Paint, whereas the figure panels were prepared using Corel Draw (Corel Photo Paint and Corel Draw, Ottawa, ON, Canada).

### Western Blot

Since neurons of the CNS express the SERT, rat brain was used as a positive control for the WB analysis for anti-SERT antibody validation. The rat brain samples utilized for the WB analysis were obtained under a separate authorized experimental protocol (authorization no. 112/2018-PR of 12 February 2018). 100 mg of rat brain and dog small intestine were homogenized in 1 mL of RIPA buffer using a high speed Ultra-Turrax homogenizer for 30 sec, after centrifugation (8000 × g), supernatants were frozen and conserved at −80°C. Total protein contents were determined by the method of Lowry using a protein assay kit (Sigma-Aldrich Co. LLC). Aliquots containing 20 μg of total proteins were separated on NuPage 4-12% bis-Tris Gel (Gibco-Life-Technologies) for 50 min at 200 V. The proteins were then electrophoretically transferred onto a nitrocellulose membrane by Turbo Blot System (Bio-Rad). The blot was washed in PBS and protein transfer was checked by staining the nitro-cellulose membranes with 0.2% Ponceau Red and the gels with Comassie Blue. Non-specific binding on nitrocellulose membranes was blocked with 5% milk powder in PBS-T20 (Phosphate Buffer Saline-0.1% Tween-20) for 1 h at RT. The membrane was then incubated over-night at 4°C with a 1:500 dilution of anti-SERT rabbit polyclonal antibody (AB9726, Merk). After several washings with PBS-T20, the membrane was incubated with the secondary biotin-conjugate antibody and then with a 1:1000 dilution of an anti-biotin horseradish peroxidase (HRP)-linked antibody. The WB was developed using a chemiluminescent substrate (Clarity Western Substrate, Bio-Rad) according to the manufacturer's instructions. The intensity of the luminescent signal of the resultant bands was determined by the ChemiDoc Instrument using Lab Image Software (Bio-Rad).

**Figure 1:**
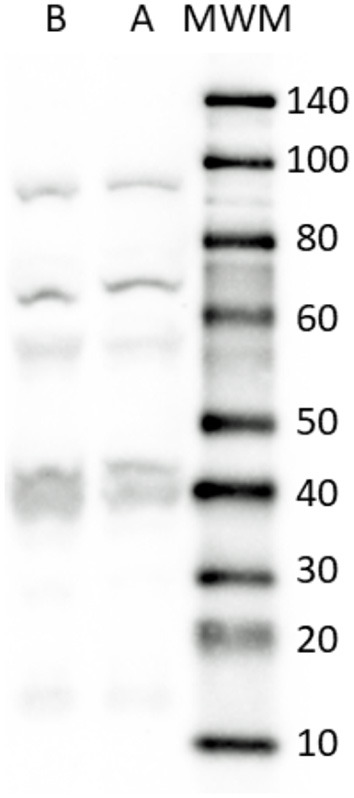
Representative image of Western Blot analysis showing the specificity of the primary antibody rabbit anti SERT in dog and rat tissues. The antibody revealed a major band close to the theoretical molecular weight of the receptor (70 kDa). In addition, bands ascribable to SERT endoproteolytic cleavage fragments were detected in all the tissues considered. Lane A, rat brain (positive control); Lane B, dog small intestine (ileum). MWM, Molecular Weight Marker. The image was slightly adjusted in brightness and contrast to match their backgrounds.

The rat brain (positive-control) and the dog small intestine revealed a band of approximately 70 kDa corresponding to the full-size SERT molecule (https://www.uniprot.org/uniprot/P31652). An additional large band (~90 kDa) representing a transporter aggregate complex and lower bands, ascribable to SERT endoproteolytic cleavage fragments, already described by other authors (52), were detectable in both samples (Figure 1).

## Results

### SERT-IR in the Dog Intestine

**Figure 2:**
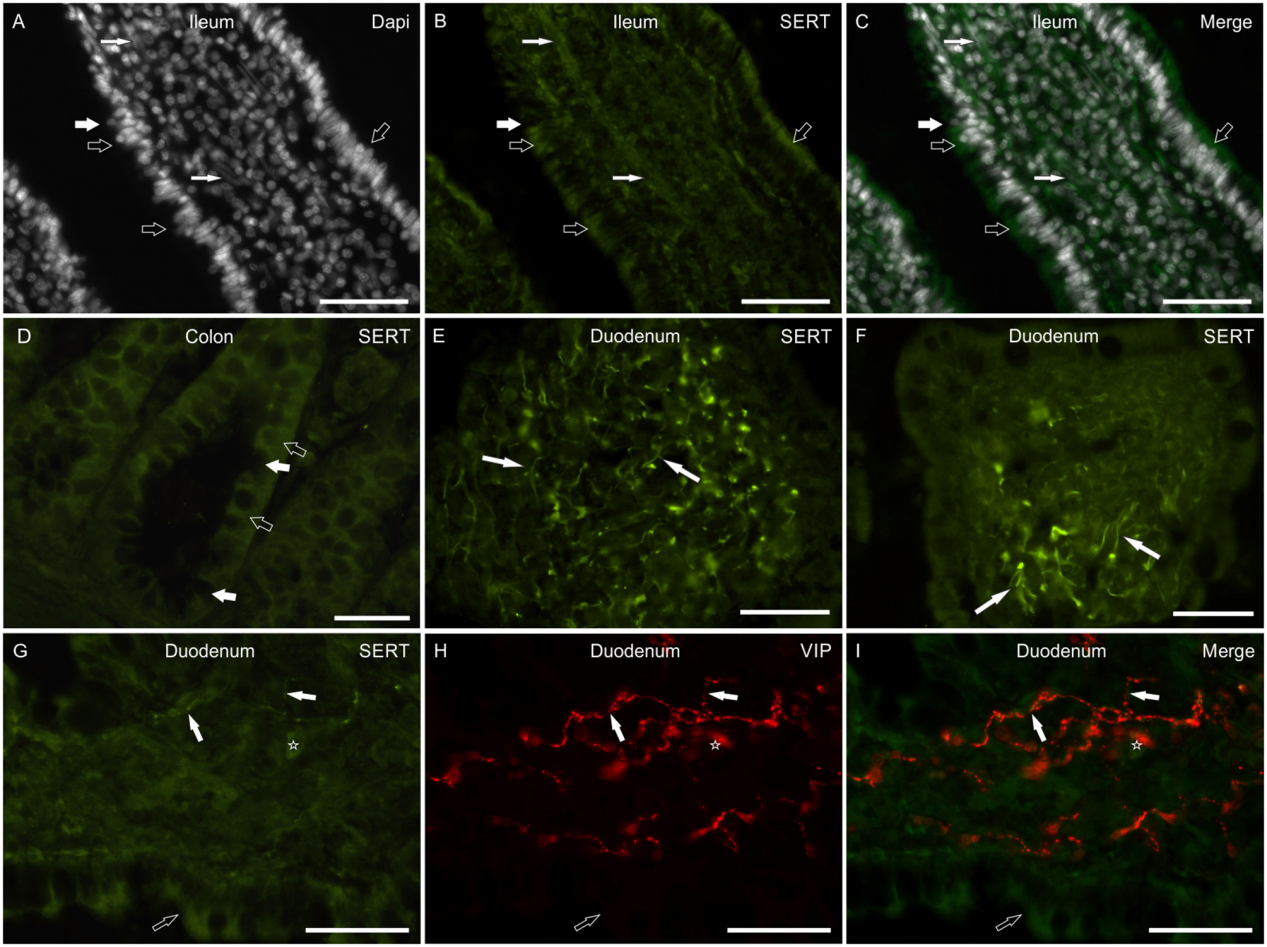
Photomicrographs of cryosections of the small and large canine intestine showing serotonin transporter (SERT) immunoreactivity (SERT-IR) in the mucosa. **(A–D)** The open arrows indicate moderate cytoplasmic SERT-IR of the epithelial cells of the ileum **(A–C)** and colon **(D)**; in the small intestine, SERT-IR was more concentrated in the apical portion of the epithelial cells. The white arrows indicate SERT negative goblet cells. The small white arrows indicate the nuclei of the smooth muscle cells which expressed faint SERT-IR. **(E,F)** The arrows indicate bright SERT immunoreactive cellular processes which were mainly visible within the apex of the villi. **(G–I)** The white arrows indicate thin SERT immunoreactive nerve processes co-expressing immunoreactivity for the vasoactive intestinal polypeptide (VIP). The open arrows indicate SERT-IR in the epithelial cells. The star indicates a lamina propria immunocyte/inflammatory cell showing SERT-IR. Scale bar: **(A–I)** 50 μm.

In the small intestine, SERT-IR was not uniformly distributed along the major axis of the villi, showing bright immunolabeling mainly in their apical parts. The epithelial cells showed SERT-IR particularly concentrated at the apical cell membrane (Figures 2A–C). In addition, SERT-IR was also visible in the intracellular compartments, close to the basolateral membrane. Moderate SERT-IR was visible in the epithelial cells of the large intestine (Figure 2D). In the small and large intestine, the goblet cells were SERT negative (Figures 2B,D). In the small intestine, the anti-SERT antibody immunolabeled thin and elongated cellular processes which were more concentrated within the apex of the villi (Figures 2E,F). Co-localization studies showed that a few of these nerve processes were VIP immunoreactive (Figures 2G–I), which is consistent with the co-expression of SERT and VIP immunoreactivity in the canine MP and submucosal plexus (SMP) neurons (see below). The anti-SERT antibody immunolabeled mucosal immunocytes/inflammatory cells (Figure 2G).

**Figure 3:**
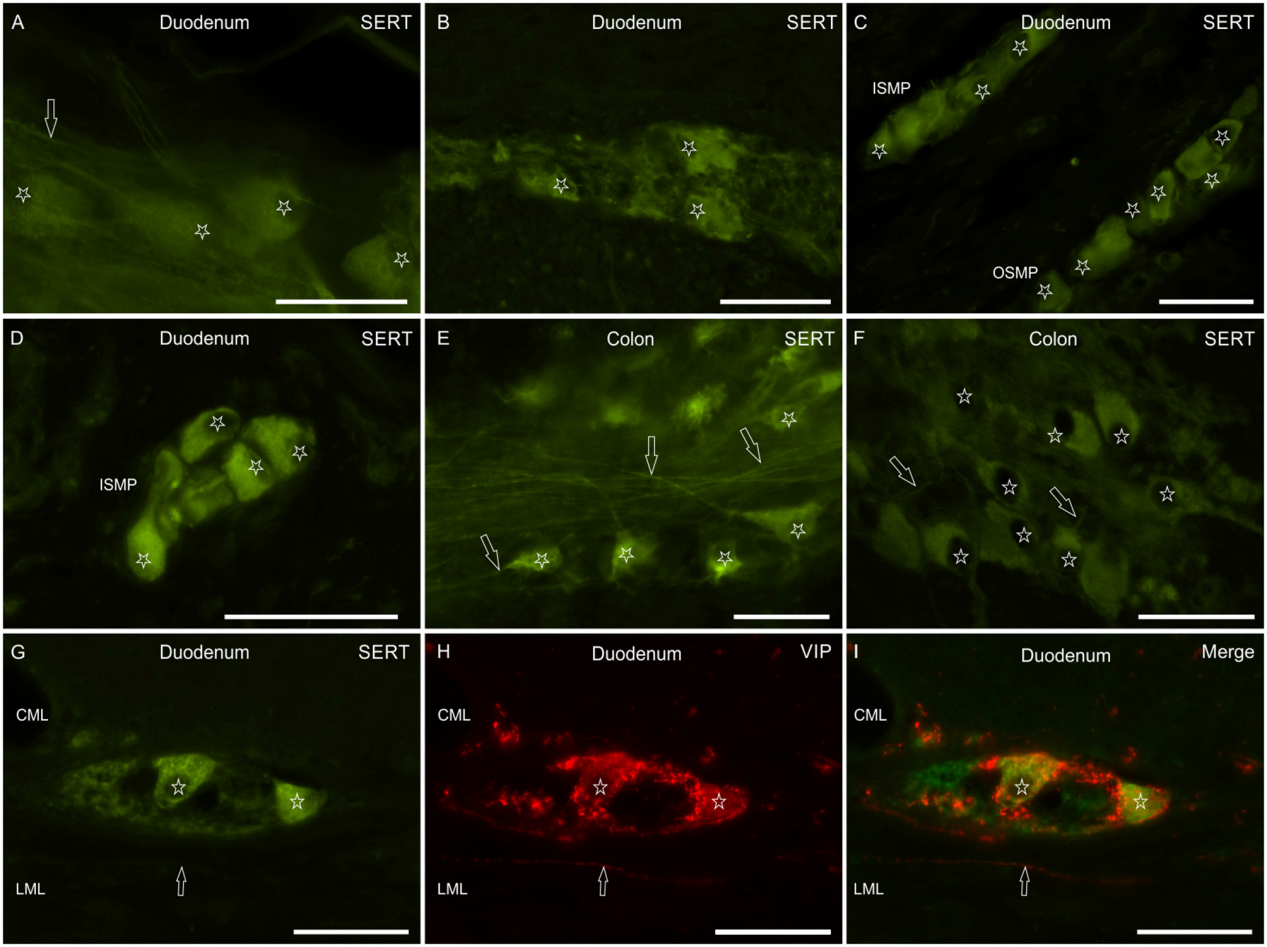
Photomicrographs showing SERT immunoreactivity (SERT-IR) in wholemount preparations **(A,E)** and cryosections **(B–D,F–I)** of the canine small and large intestine. **(A–F)** The stars indicate myenteric **(A,B,E,F)** and submucosal **(C,D)** plexus neurons showing SERT-IR; in the submucosa, SERT-IR neurons were seen in the inner (ISMP) and outer (OSMP) portions of the submucosal plexus. The arrows indicate SERT immunoreactive nerve processes arising from the enteric neurons. **(G–I)** The stars indicate two SERT immunoreactive myenteric plexus neurons **(G)** co-expressing immunoreactivity for the vasoactive intestinal polypeptide (VIP) **(H)**. The arrow indicates a VIP immunoreactive nerve fiber. CML, circular muscle layer; LML, longitudinal muscle layer. Scale bar: **(A–I)** 50 μm.

Myenteric and submucosal neurons showed bright cytoplasmic SERT-IR (Figures 3A–F). Co-localization studies indicated that some of the MP and SMP neurons co-expressed VIP immunoreactivity (Figures 3G–I).

The anti-SERT antiserum immunolabeled the smooth muscle cells of the *tunica muscularis*, which expressed faint-to-moderate SERT-IR, and also thin fascicles of smooth muscle cells within the central axis of the villi (Figures 2A–C).

### SERT-IR in the Rat Intestine (Positive Control)

**Figure 4:**
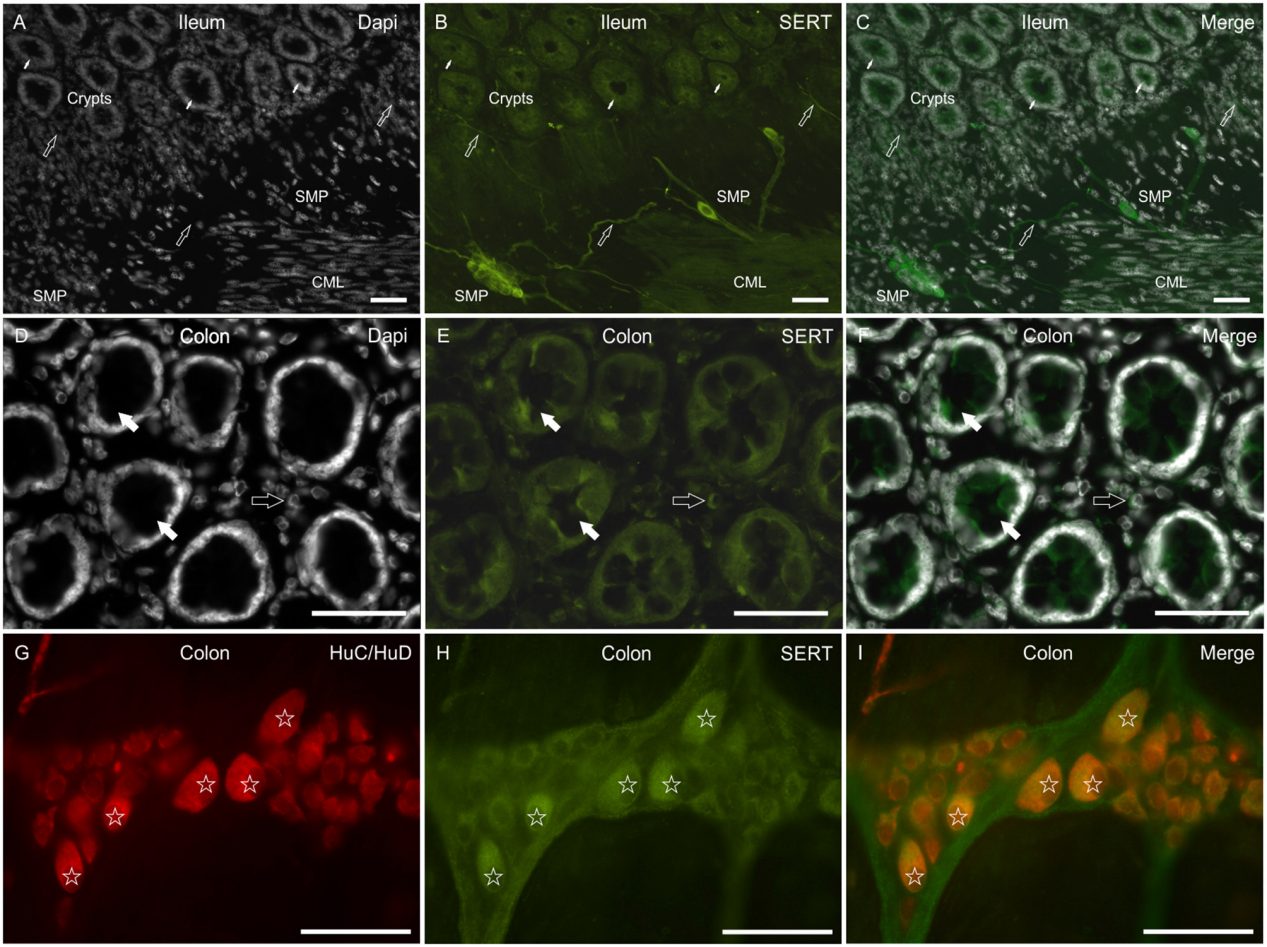
Photomicrographs showing SERT immunoreactivity (SERT-IR) in cryosections **(A–F)** and wholemount preparations **(G–I)** of the rat small and large intestine. **(A–C)** The small arrows indicate some crypts in which the epithelial cells of the ileum show faint-to-moderate SERT-IR. The open arrows indicate mucosal and submucosal SERT immunoreactive nerve processes arising from the submucosal plexus (SMP) neurons, which showed bright SERT-IR. Faint-to-moderate SERT-IR is also expressed by the smooth muscle of the *tunica muscularis*. **(D–F)** The open arrows indicate SERT negative goblet cells of the rat colon. The open arrows indicate lamina propria immunocytes/inflammatory cells expressing moderate SERT-IR. **(G–I)** The stars indicate some myenteric plexus neurons co-expressing bright HuC/HuD and SERT-IR. CML, circular muscle layer. Scale bar: **(A–C)** 100 μm; **(D–I)** 50 μm.

The anti-SERT antibody immunolabeled the enterocytes of the small and large intestine, which showed faint-to-moderate cytoplasmic SERT-IR (Figures 4A–F). In the lamina propria, the antibody immunolabeled a rich network of nerve fibers arising from SERT immunoreactive SMP neurons (Figures 4B,C). A number of *lamina propria* cells (likely immunocytes/inflammatory cells) showed SERT-IR (Figure 4E). As seen in the dog, MP and SMP enteric neurons expressed bright cytoplasmic SERT-IR (Figures 4G–I). Faint-to-moderate SERT-IR was also expressed by the smooth muscle cells of the *tunica muscularis* (Figure 4B).

### SERT-IR in the Human Intestine

**Figure 5:**
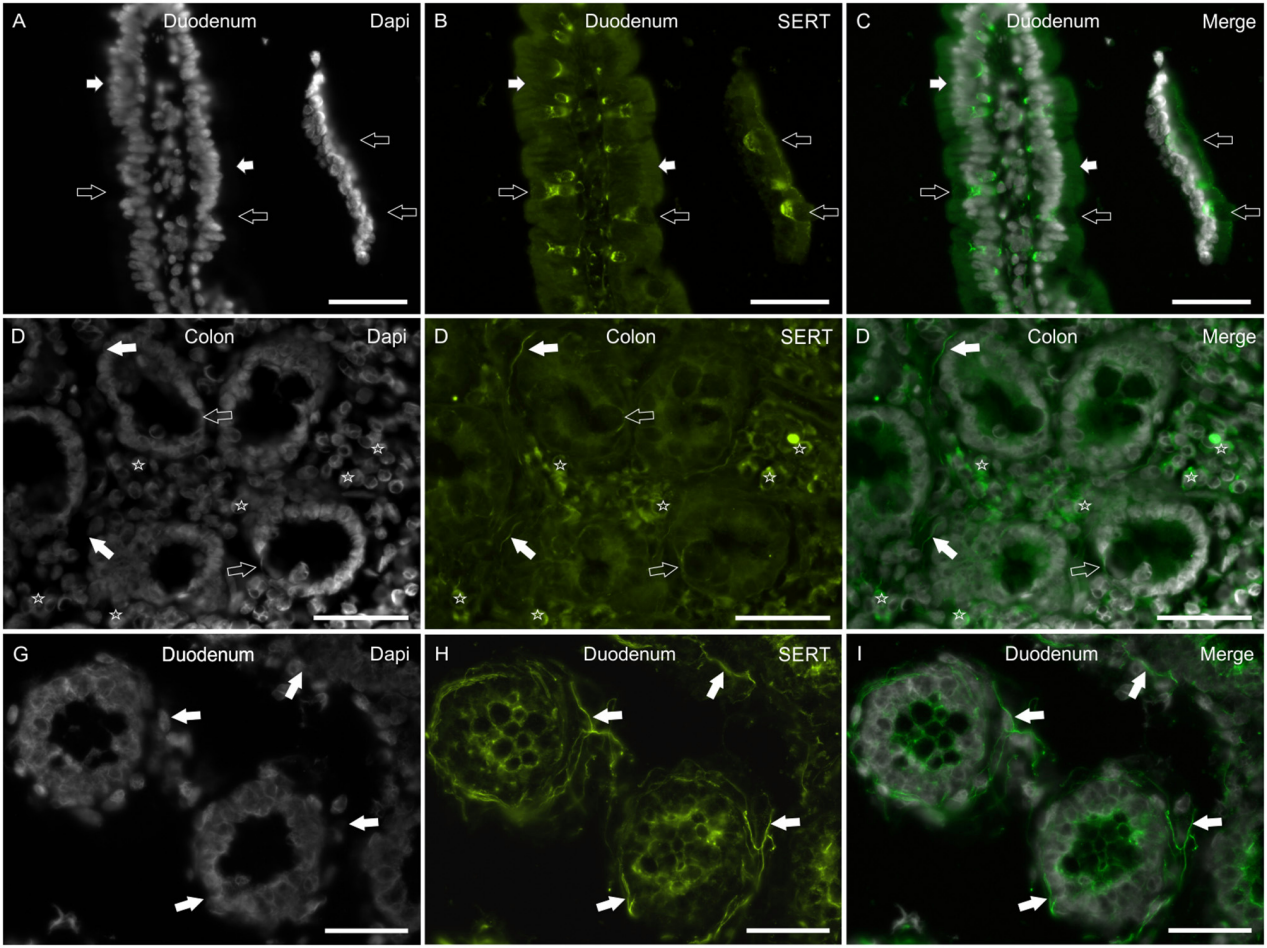
Photomicrographs showing SERT immunoreactivity (SERT-IR) in cryosections of the human duodenum **(A–C,G–I)** and colon **(D–F)**. **(A–C)** The white arrows indicate cytoplasmic SERT-IR **(B)** which is more concentrated in the apical part of the enterocytes of the human duodenum. The open arrows indicate goblet cells showing bright SERT-IR in the basal portion of the cells. **(D–F)** The open arrows indicate goblet cells of the human colon showing SERT-IR in the basal portion of the cells and in the cell membrane **(D)**. The white arrows indicate SERT-IR nerve processes. The stars indicate some lamina propria immunocytes/inflammatory cells co-expressing SERT-IR. **(G–I)** The arrows indicate bright SERT immunoreactive nerve processes which encircle the crypts of the human duodenum. Scale bar: **(A–I)** 50 μm.

In the mucosa of the small and large intestine, the anti-SERT antibody immunolabeled the cytoplasm of the epithelial cells; SERT-IR was more concentrated in the upper portions of the cells (Figures 5A–F). It is worth noting that the anti-SERT antibody brightly immunolabeled the cytoplasm and cell membrane of the goblet cells of the small and large intestine (Figures 5A–F).

As seen in the rat (and less in the dog) intestine, the anti-SERT antibody immunolabeled an impressive network of nerve fibers encircling the crypts (Figures 5G–I).

The SERT-IR was expressed by lamina propria immunocytes/inflammatory cells also in the human intestine (Figures 5D–F).

**Figure 6:**
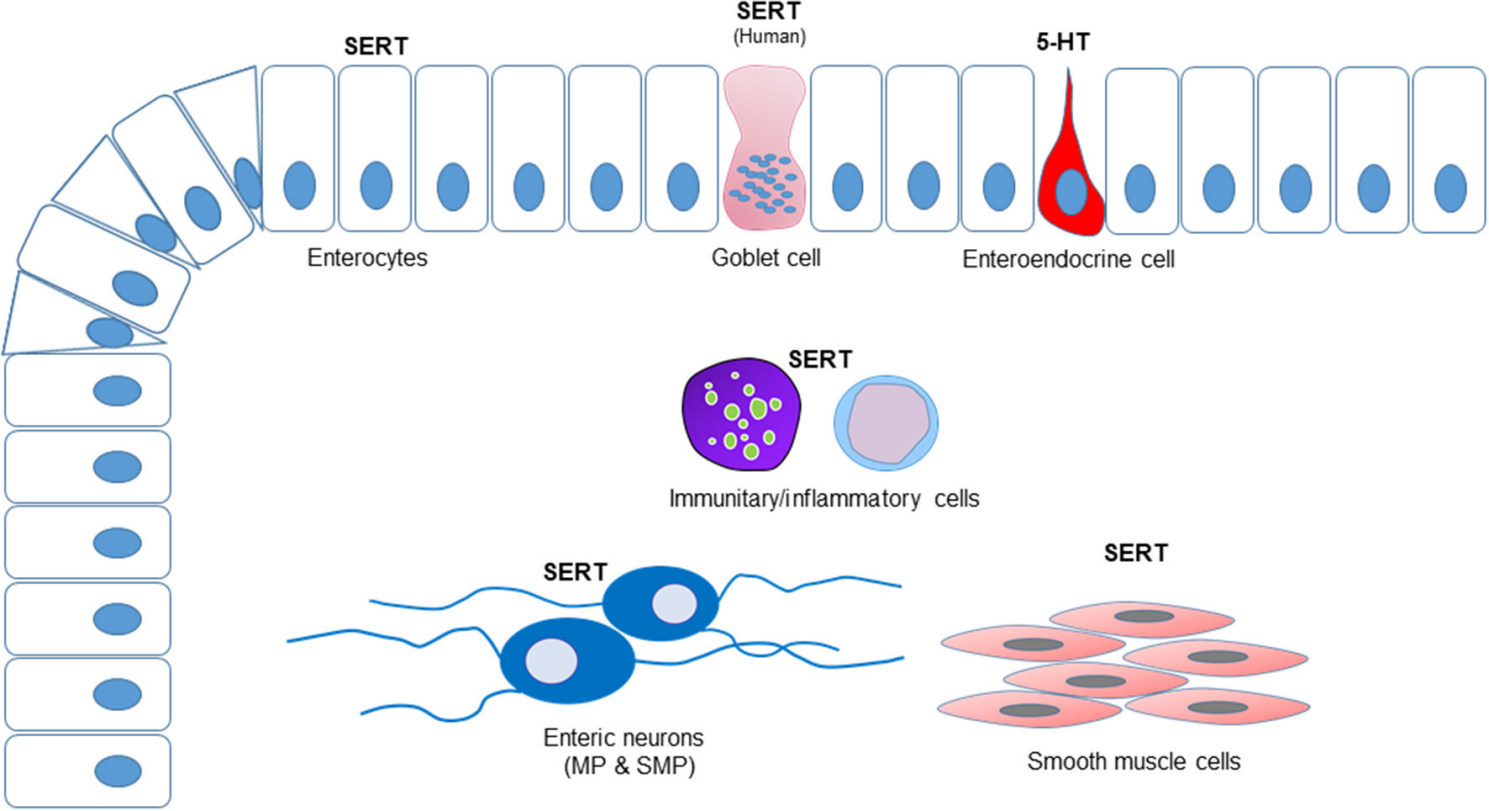
Graphical representation of the distribution of the serotonin transporter immunoreactivity (SERT-IR) in the canine, rat, and human intestines. The enterocytes expressed SERT-IR in all the species considered whereas only in the human intestine SERT-IR was observed in the goblet cells. Serotonin (5-HT) immunoreactivity was expressed by the enteroendocrine cells of the dog, but not by the enteric neurons. In all the species considered, a number of lamina propria cells (immunocytes/inflammatory cells) expressed SERT-IR. Serotonin transporter-IR was observed in the enteric neurons. The smooth muscle cells of the *tunica muscularis* of the canine and rat intestines expressed SERT-IR; in the present study, the submucosa, the *tunica muscularis* and the myenteric plexus neurons of the human intestine were not available.

The results of the cellular distribution and intensity of the SERT immunolabeling of the anti-SERT antibody in the canine, rat and human intestines are summarized in semiquantitative (Table 4). The qualitative distribution of the SERT-IR within the canine, rat and human intestines are graphically depicted (Figure 6).

**Table 4:** Semiquantitative evaluation of the density of serotonin transport (SERT) immunoreactivity in different cellular elements (epithelium, lamina propria cells, enteric neurons, *tunica muscularis*) of the dog, rat, and human intestine.

**Species**	**Epithelium**	**Enteric neurons**	**Lamina propria cells**	***Tunica muscularis***
Dog	C+/++	C++ (MP) C++ (SMP)	C+/++	C+
Rat	C+/++	C++ (MP) C++ (SMP)	C^++^	C+
Human	C++ GC+++	NA	C++/+++	NA

*The immunoreactive cells were graded as: –, negative; +, faintly stained; ++, moderately stained; +++, brightly stained*.

*C, cytoplasmic; GC, goblet cells; LML, longitudinal muscle layer; MP, myenteric plexus; N, nuclear; NA, not available; SMP, submucosal plexus*.

### Serotonin-IR in the Dog Intestine

No 5-HT immunoreactive neurons and nerve fibers were observed in tangential (to the serosa) or vertical cryosections, or in the wholemount preparations of the small and large intestine. On the contrary, 5-HT-IR was brightly expressed by mucosal ECs (Supplementary Figures 1A–C) which were more concentrated in the bottom of the crypts.

## Discussion

### SERT on the Enterocytes

Serotonin is produced by mucosal ECs and secreted into the lumen and lamina propria under different types of stimuli, such as mucosal stroking, ingested chemicals (e.g., tastants, short chain fatty acids or toxins), GIT contraction and commensal organisms. Enterochromaffin cells have a direct connection with nerve fibers (7, 23, 53, 54); 5-HT secreted into the lumen by the ECs adds to the 5-HT produced by the gut microbiota or 5-HT from alimentary sources (55, 56). When secreted into the lumen, 5-HT regulates secretory processes (55); when released within the lamina propria, 5-HT activates extrinsic and intrinsic sensory fibers. The rich sensory innervation of the GIT mucosa is not the only target of 5-HT produced by the ECs. In fact, other non-neuronal GIT cells, such as epithelial cells, platelets, endothelial cells and immunocytes/inflammatory cells express 5-HT receptors and are therefore influenced by 5-HT regulation/control (7, 11, 57, 58) (see below). If, in addition to sensory nerve fibers, the targets of 5-HT secreted by the ECs are other types of mucosal cells, it can be postulated that 5-HT plays a role as a mucosal signaling molecule, which must account for mucosal 5-HT inactivation (28). Significantly, the SERT should be present on all cells targeted by 5-HT. It is also plausible to consider that the notable number and diversity of mucosal SERT immunoreactive cells may represent a second line of defense, inactivating the 5-HT which has escaped neuronal reuptake, thus preventing uncontrolled spreading of the 5-HT signal. In the present study, the expression of the SERT by MP and SMP neurons, immunocytes/inflammatory cells, and smooth muscle cells was highlighted whereas the expression of the SERT by epithelial cells was somewhat reduced. In the dog, rat and human tissues, the epithelial SERT immunolabeling was faint-to-moderate and was predominantly localized to the intracellular compartments, although it was also observed in the apical cell membrane. The distribution of the SERT-IR observed in the epithelium of the dog and rat was consistent with what has been observed in the rat by Wade et al. (27) while the SERT-IR expressed by the epithelium of the human intestine was consistent to that observed by other authors (30–32). Serotonin transporter-IR was faint in the epithelium of the dog colon; this finding was consistent with that obtained by Gill et al. (30) on human tissue; they observed regional differences of the SERT (mRNA and protein levels) with the highest expression in the small intestine (ileum>duodenum>jejunum) and the lowest (almost absent) in the colon. This finding might indicate the presence of alternate mechanisms of 5-HT transport by means of related monoamine transporters (30, 59) or the expression of a spliced variant of the SERT. The intracellular concentration of SERT-IR reflects the location of an intracellular pool of transporter molecules which can be recruited to membranes. The expression of SERT-IR on the tips of the enterocytes makes luminal 5-HT reuptake possible while its basolateral expression suggests 5-HT reuptake from the lamina propria (28).

In the human duodenum, the anti-SERT antibody brightly immunolabeled the goblet cells whereas it failed to identify these cells in the mucosa of the rat and dog intestine; this never before reported evidence could represent a datum of some relevance since the use of drugs which act on the SERT could be correlated to a variation in the secretory processes of the intestine. The Authors findings were consistent with those reported by Hoffman et al. (60) who localized the 5-HT4 receptor on goblet cells of the human intestine and showed that stimulation of this receptor evoked goblet cell degranulation. On the contrary, Kaji et al. (55) did not identify this receptor on goblet cells of the rat intestine.

### SERT on Enteric Neurons

It is known that the SERT is expressed on the cell membranes of serotoninergic neurons, and that it is necessary for the introduction/reuptake of 5-HT within the cell where enzymes (monoamine oxidase or glucuronyl transferase) take part in 5-HT degradation/catabolism (26, 28). In the dog intestine, the presence of serotoninergic neurons is controversial; while Björck et al. (61) described positive nerve cell bodies in the MP and SMP of the canine small intestine, Mann and Bell (62), failed to identify serotoninergic neurons in the ileum. In the majority of the species studied, serotonergic neurons, which are considered as descending interneurons (5), represent only 1% of the myenteric neurons (13, 63). The findings in the present study regarding dogs are partially consistent with those of Mann and Bell (62); in fact, neither 5-HT positive neurons nor nerve fibers, which were instead observed by Mann and Bell (62) were observed in the present study. However, despite the absence of serotoninergic neurons, a significant number of canine enteric neurons expressed bright SERT-IR. It is plausible to consider that canine enteric neurons, which express 5-HT4 serotoninergic receptors (personal observation of Dr. Chiocchetti), may reach the mucosa and may be affected by the serotonin released by the ECs. The notable number of rat enteric neurons expressing SERT-IR also indicated that not only serotoninergic neurons can express the serotonin reuptake transporter. Glatzle et al. (64) showed that different subclasses of rat enteric neurons express the 5-HT3 receptor, such as cholinergic and VIPergic neurons. In the present study, canine VIPergic neurons and fibers co-expressing SERT-IR were observed. Glatzle et al. (64) showed that the nerve fibers immunoreactive for the 5-HT3 receptor encircled the rat intestinal crypts; the latter evidence reinforces the presence of the SERT immunoreactive nerve fibers observed in the present study around the crypts of rat and human intestine.

### SERT on Immunocytes/Inflammatory Cells

In recent years, a number of immunoregulatory functions have been ascribed to 5-HT. The Authors observed numerous lamina propria immunocytes/inflammatory cells showing SERT immunolabeling in all the species considered. The findings in the present study were consistent with the evidence of SERT expression in B and T lymphocytes, dendritic cells, macrophages and mast cells in human and murine species (11, 65–68). However, in the present study, the phenotype of the numerous immune cells of the lamina propria expressing SERT-IR was not investigated. Although it has been reported that platelets play a central role in delivering 5-HT to inflammatory effector cells, the close proximity of 5-HT-producing ECs and lamina propria immunocytes/inflammatory cells suggests a direct action of 5-HT on these cells and may justify the high density of SERT-IR observed in the present study.

### SERT on Smooth Muscle Cells

Serotonin transporter-IR was observed in the smooth muscle cells of the *tunica muscularis* of dogs and rats, and of the *muscularis mucosae* of dogs and humans. The finding in the present study was consistent with the evidence of SERT expression in the colonic smooth muscles of the rat (69). Since only mucosal tissues of the human intestine were utilized in the present study, the findings obtained in dogs and rats cannot be confirmed in this species. However, in human intestinal smooth muscle cells, it has been shown that 5-HT receptors mediate contraction (5-HT2a) and relaxation (5-HT4) (70). In the esophagus of *Suncus murinus*, 5-HT induces contractile responses of the longitudinal layer of the *muscularis mucosae* which are mediated via 5-HT1 and 5-HT2 receptors on smooth muscle cells (71). The 5-HT2b receptor has been identified in the muscle cells of the rat and the mouse stomach, and in the human intestine (72, 73). Thus, 5-HT seems to be able to directly contribute to peristalsis; therefore, it is also plausible that smooth muscle cells express a 5-HT reuptake system. It has been shown that fluoxetine, which acts by inhibiting serotonin reuptake, may have direct effects on the smooth muscle in humans and dogs (74). In humans, up to 30% of patients taking fluoxetine experience gastrointestinal side effects, most commonly nausea and diarrhea (75). This side effect should be considered in dogs receiving fluoxetine therapy.

## Limitations

The Authors acknowledge some limitations which should be taken into consideration when interpreting the results of this study since many factors could potentially alter the SERT expression in the tissues. The underlying pathological conditions of the dogs in the study and the medications received could have influenced the SERT expression in the gut (76). A different dietary supplementation of tryptophan could alter the serum level of serotonin and might modulate the intestinal SERT expression should also be considered (77). Intestinal microbiota can also change the level of the SERT and regulate gastrointestinal function (78), and intestinal dysbiosis could upregulate the SERT expression (37). It is not known whether breed-related differences could also determine a modification in SERT expression in dogs. The age and the sex of the dogs could also potentially influence the expression of the SERT, as shown in the ileum of young guinea pigs (79, 80), and in the central nervous system of female rats (81), respectively. The limited number of dogs considered in the current study, the reduced representation of male and female dogs, as well as adult and young dogs, represent another limitation of the study and makes it difficult to establish any differences in immunoreactivity for the SERT in these categories of animals (male *vs*. female; adult *vs*. young).

## Conclusion

The present study is the first immunohistochemical demonstration of SERT-IR in the dog intestine, and provides additional anatomical evidence regarding the SERT distribution in the rat and the human intestines. The majority of the 5-HT produced by the body is synthesized in the intestine; thus it seems plausible that there are several diffuse cellular elements in the intestine which can remove excess 5-HT. Collectively, it has been shown in this study that epithelial cells, lamina propria immunocytes/inflammatory cells, enteric neurons, and smooth muscle cells also express the SERT in the intestines of dogs, rats and humans, and may exert a role in 5-HT reuptake. In order to better interpret the possible occurrence of side effects, as has been observed in human medicine, these data should be taken into consideration when using SERT inhibitor drugs in dogs (fluoxetine). In addition, the expression of SERT-IR on human goblet cells was highlighted. Finally, the present study indicated that the canine ENS lacked serotoninergic neurons.

## Data Availability Statement

The raw data supporting the conclusions of this article will be made available by the authors, without undue reservation.

## Ethics Statement

Ethical review and approval was not required for the study on human participants in accordance with the local legislation and institutional requirements. The patients/participants provided their written informed consent to participate in this study. The animal study was reviewed and approved by Rat: authorization no. 112/2018-PR of 12 February 2018. Written informed consent for participation was not obtained from the owners because Dogs died naturally or were euthanized for humane reasons. According to Directive 2010/63/EU of the European Parliament and of the Council of 22 September 2010 regarding the protection of animals used for scientific purposes, Italian legislation (D. Lgs. n. 26/2014) does not require any approval by competent authorities or ethics committees since this study did not induce any pain or influence any therapeutic decisions.

## Author Contributions

RC and MP designed the study. GG, FG, CT, and RC performed the immunohistochemical experiments. CB and MF carried out the Western Blot analysis. GG, MP, and RC collected the material in compliance with ethical guidelines and performed part of the experiments. RC and GG quantified the data. RC and FG analyzed the data and wrote the manuscript. All authors contributed to the article and approved the submitted version.

## Funding

This work was funded by the University of Bologna (Ricerca Fondamentale Orientata grant).

## Conflict of Interest

The authors declare that the research was conducted in the absence of any commercial or financial relationships that could be construed as a potential conflict of interest.

## Publisher's Note

All claims expressed in this article are solely those of the authors and do not necessarily represent those of their affiliated organizations, or those of the publisher, the editors and the reviewers. Any product that may be evaluated in this article, or claim that may be made by its manufacturer, is not guaranteed or endorsed by the publisher.

## Supplementary Material

Supplementary Figure 1 Immunolabeling of the enteroendocrine cells (arrows) of the canine colon using a monoclonal antibody against serotonin (5-HT).
