# Supplementary figures and images for: Localization of the Serotonin Transporter in the Dog Intestine and Comparison to the Rat and Human Intestines
Source: Front Vet Sci. 2022 Jan 5;8:802479. doi: 10.3389/fvets.2021.802479 (PMC8766808; doi:10.3389/fvets.2021.802479)

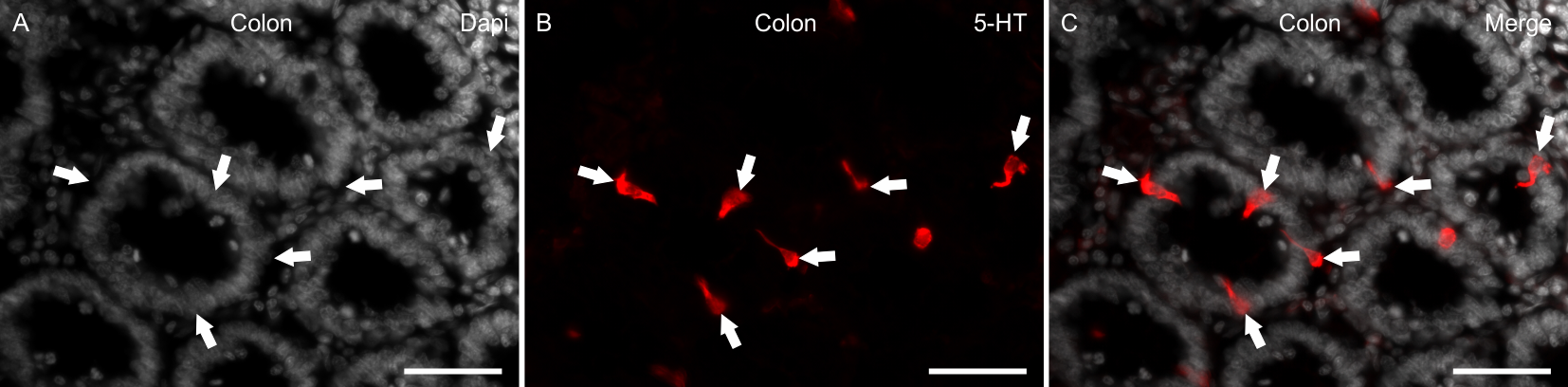

Supplement: Supplementary Figure 1 — Immunolabeling of the enteroendocrine cells (arrows) of the canine colon using a monoclonal antibody against serotonin (5-HT). [file Image_1.TIF]
